# Use of Fish Skin Graft in Management of Combat Injuries Following Military Drone Assaults in Field-Like Hospital Conditions

**DOI:** 10.1093/milmed/usad028

**Published:** 2023-02-13

**Authors:** Fouad Reda, Hilmar Kjartansson, Steven L A Jeffery

**Affiliations:** Ajapnyak Medical Center, Yerevan 0038, Armenia; Kerecis LLC, Staff Specialist Landspitali University Hospital Reykjavik Iceland, Reykjavic, Iceland; 202 Field Hospital, Birmingham B14 6NY, UK

## Abstract

**Introduction:**

The 2020 Nagorno-Karabakh war was an armed conflict between Azerbaijan and Armenia over an ethnically and historically significant region. This manuscript is a report on the forward deployment of acellular fish skin graft (FSG) from Kerecis™, a biologic, acellular matrix derived from the skin of wild-caught Atlantic cod that contains intact epidermis and dermis layers. The usual intention of treatment under adverse circumstances is to temporize wounds until better treatment can be attained, although ideally, rapid coverage and treatment are necessary to prevent long-term complications and loss of life and limb. An austere environment, such as the one experienced during the conflict described here, presents considerable logistical barriers for the treatment of wounded soldiers.

**Materials and Methods:**

Dr H. Kjartansson from Iceland and Dr S. Jeffery from the United Kingdom traveled to Yerevan, near the heart of the conflict, to deliver and train on using FSG in wound management. The primary goal was to use FSG in patients where stabilization and improvement in the wound bed were needed before skin grafting. Other goals were to improve healing time, achieve earlier skin grafting, and have better cosmetic outcomes upon healing.

**Results:**

Over the course of two trips, several patients were managed with fish skin. Injuries included large-area full-thickness burn and blast injuries. Management with FSG induced wound granulation several days sooner in all cases, and even weeks in some instances, allowing a stepdown in the reconstruction ladder with earlier skin grafting procedures and a reduction in requirement of flap surgery.

**Conclusions:**

This manuscript describes a successful first instance of forward deployment of FSGs to an austere environment. FSG, in this military context, has shown great portability, with easy transfer of knowledge. More importantly, management with fish skin has shown faster granulation rates in burn wounds for skin grafting, resulting in improved patient outcomes with no documented infections.

## INTRODUCTION

The 2020 Nagorno-Karabakh War was an armed conflict between Azerbaijan and Armenia over an ethnically and historically significant region. The war started on September 27, 2020, and lasted until a ceasefire on November 10, 2020. The war was notorious for the use of drones, both for reconnaissance and for targeted airstrikes, as well as the use of long-range weapons and sensors. This has heralded a new age of warfare.

The ammunition used for drone combat is unclear; however, there were reports of the use of white phosphorous compounds. White phosphorus is prevalent in many combustibles used by military forces.^1^ Exposure to this substance can cause fatal chemical burns and can be the source of significant morbidity and lengthy hospital stays.^1^ These burns mainly affect exposed areas of the skin, such as the face and hands. This new style of drone warfare has resulted in more burn and flash burn injuries than those previously seen in traditional combat. Blast and gunshot injuries were also well-reported in this conflict. Blast injuries consist of multiple types of trauma, including thermal and chemical burns.^2^

Both sides suffered many casualties during the war, and Armenia’s military hospitals were overwhelmed with the number of injured during the conflict. As such, civilian hospitals started to be utilized to treat soldiers. The overflow of wounded soldiers was transported directly to capital hospital facilities from the field. Nevertheless, the hospital system was overwhelmed, making early surgery impossible. In many instances, patients would have to wait upward of a week for surgery. During that time, burns were managed with regular dressing changes, and severe pain was controlled with high-dose opiates. Most of the burns seen coming from the battlefield were deep-partial or full-thickness burns.

Total excision of burn wounds should be the current standard procedure, as partial excision of the burn area postsurgery leads to common infections, resulting in treatment with antibiotics and/or the need for further surgery. Unfortunately, during the conflict, many burn wounds were treated by partial excision, which prompted exploration of an innovative solution for treating these wounds.

The dire situation arising from the Nagorno-Karabakh War prompted a member of parliament from the Armenian government to reach out to Iceland (Kerecis) as the two countries share historical ties. Within 72 hours, plans for the shipping of product and deployment of two physicians, Dr H. Kjartansson from Iceland and Dr S. Jeffery from the United Kingdom, were made. Both physicians traveled to Yerevan, near the heart of the conflict, to deliver and train on using FSG in wound management. The primary goal was to use FSG in patients where stabilization and improvement in the wound bed were needed before skin grafting. Other goals were to improve healing time, achieve earlier skin grafting, and have better cosmetic outcomes upon healing.

## METHODS

This manuscript is a report on the forward deployment of acellular fish skin graft (FSG) from Kerecis™ (Kerecis Omega3, Kerecis, Isafjordur, Iceland), a biologic, acellular matrix derived from the skin of wild-caught Atlantic cod that contains intact epidermis and dermis layers. Due to gentle processing, the matrix retains natural Omega3 fatty acids.^3^ The fish skin product has a 3-year shelf life at its ideal storage at room temperature in a dry environment.^4^ The fish skin is reconstituted using only saline.

FSG comes in the form of a thick sheet (average thickness 0.61 mm ± 0.15 mm [*N* = 50; Kerecis internal report]) and is easy to handle with minimal training required for use. Its relative thickness shields against environmental irritants, reducing infection risk in much the same way as dressings. Furthermore, FSG possesses intact Omega3 fatty acids, which may play a role.^3,5,6^ The literature has demonstrated bacteriostatic and anti-inflammatory properties of fatty acids.^3,5,7–9^ In addition, the product is easy to use and adapts well to irregular surfaces. It is provided in sheets available premeshed and up to 300 cm^2^ in size. After hydration, it can stretch to cover up to 540 cm^2^. Large sheets require fewer joints and less patching in an application. It is usually kept in place with the use of staples, with either negative pressure wound therapy (NPWT) or a bolster dressing. Efficacy was assessed according to whether the wound was suitably prepared for skin grafting. The dressings were changed every 3-5 days after the surgery.

The usual intention of treatment under adverse circumstances is to temporize wounds until better treatment can be attained, although ideally, rapid coverage and treatment are necessary to prevent long-term complications and loss of life and limb.

## RESULTS

Over the course of two trips, several patients were managed with fish skin. Injuries included large-area full-thickness burn and blast injuries. Patients were selected consecutively while the authors were in that particular hospital. The majority of the wounds were 3- to 5-days old and had had one initial debridement and simple wet-to-dry dressings before the first application of FSG. When necessary, the wounds received further debridement. FSG was applied followed by NPWT, and follow-up was performed 7 days later. At follow-up, it was assessed whether the patient was ready for skin grafting.

Management with FSG induced wound granulation several days sooner in all cases and even weeks in some instances, allowing a stepdown in the reconstruction ladder with earlier skin grafting procedures and a reduction in the requirement of flap surgery. No infections were reported in any of the cases where FSG was used. Improvement in pain levels was not assessed. The physicians went to four other hospitals in the region to train surgeons in the use and application of FSG. Training was adopted readily, as FSG has a simple reconstitution and application process making it easy to use. In the following, we describe three cases involving large blast injuries and burns where FSG was used to treat the wounds.

### Case 1

**FIGURE 1.**
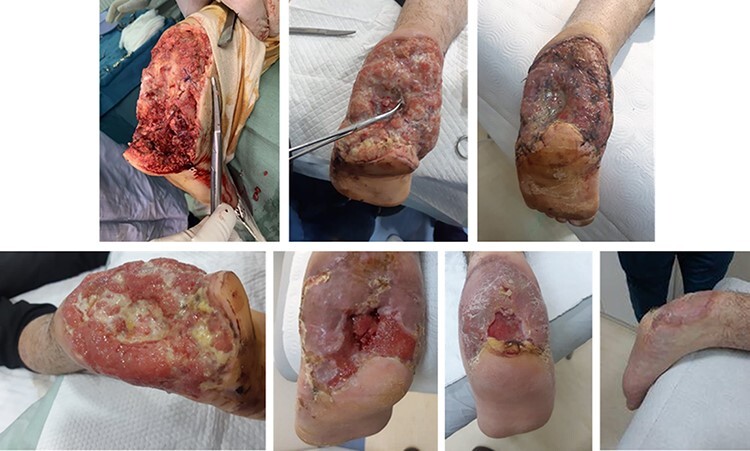
A 19-year-old male with full-thickness blast injury down to bone on left foot. Nonviable tissue was debrided, and fish skin graft was applied, used in combination with NPWT.

Case 1 was a 19-year-old male with a large-area full-thickness injury, including torso burn, as a result of a blast injury (Fig. 1). One of the injuries from the blast was a large wound on the heel of the left foot, with exposed bone. FSG was used in combination with NPWT. By the time FSG was applied, the majority of wounds were 8 days old but had received initial debridement before the first application of FSG.

### Case 2

**FIGURE 2.**
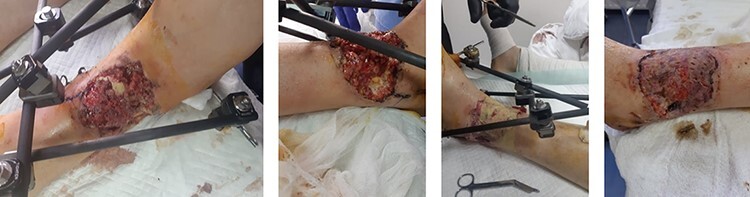
A 32-year-old male with lower right tibial fracture accompanied by a large open wound. The fracture was stabilized, the wound debrided before being temporized with FSG, and then skin graft was applied.

Case 2 is a 32-year-old soldier with a right lower tibial bone fracture. The patient presented with an open fracture, and an external fixator was inserted to stabilize the fracture. A large open wound (15 cm × 21 cm in area) accompanied the fracture. The wound was debrided three times to ensure nonviable tissue, and debris was removed before FSG was applied. The wound was followed up every 3 days with new dressings added. Overall, three applications of FSG were performed before the wound bed being ready for split-thickness skin grafting. Figure 2 shows the initial wound presentation, application of FSG, and eventual covering with split-thickness skin graft.

### Case 3

**FIGURE 3.**
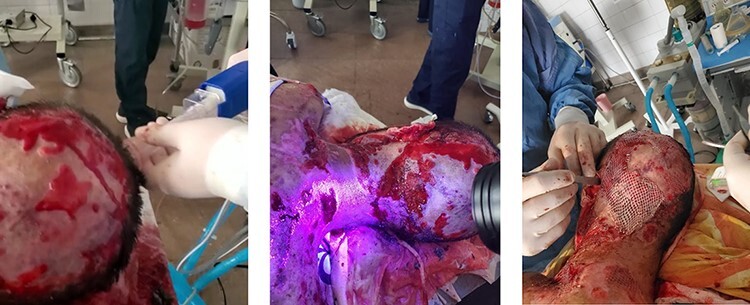
A 28-year-old male with 75% TBSA burn wound following a blast injury. Water jet pressure cleaning was performed before wound debridement, and grafting with fish skin graft was made.

The third case presented here was a 28-year-old male with a full body burn caused by an explosion containing white phosphorus (Fig. 3). The Total Body Surface Area affected was around 75%. Overall, 10 debridements were performed after water jet pressure cleaning. FSG was used as a substitute to skin grafting, and the process was successful.

## DISCUSSION

An austere environment, such as the one experienced during the conflict described here, presents considerable logistical barriers for the treatment of wounded soldiers. Regarding wound or burn treatment, ultra-low cold storage of allograft is infrequently available, meaning that alternative methods are required. Field treatment options need to be as close as possible to shelf stable in a multitude of environments and climates. FSG presents favorable characteristics for use in such austere environments: the product is rugged and robust, is low weight, is easy to transport, and requires no complex tools or equipment for use other than standard field medical equipment. Furthermore, the minimal training required for use makes FSG an ideal product for use when an overwhelmingly number of patients may need rapid treatment.

The application of FSG achieves early granulation tissue formation and a good coverage of the underlying wound, resulting in low infection risk.^3^ However, as opposed to dressings or other temporizing measures, applying FSG begins the wound-healing process rather than simply buying time.^10,11^ The presence of omega-3 fatty acids in the fish skin may have an effect on inflammation,^8,9^ which allows for better revascularization of the wound bed allowing more favorable conditions to take on allograft (although FSG itself is sufficient for healing in some wounds).^7^ FSG has been shown previously to reduce the engraftment time in pediatric patients by a half.^12^

As long as there is access to sterile debridement tools, sterile saline, and room temperature dry storage, FSG can be used. The case reports presented here show real-world outcomes of utilizing these logistically advantageous properties under such demanding conditions.

Nonetheless, use on wounded veterans under such austere conditions does represent an uncommon use of the product and certain aspects of these case reports must be acknowledged. First, this was not a controlled study, and given the nature of the patients presented, there was no documented assessment of the wound bed before FSG application, nor extensive follow-up. Second, NPWT was used in conjunction with FSG (which is a standard and well-accepted procedure for treating surgical wounds with FSG), which makes true evaluation of the effectiveness of FSG in such patients difficult. However, it should be noted that FSG is a CE-marked and FDA-approved product (several products are available in the Kerecis Omega3 series), and therefore, its use in patients, such as those described in this manuscript, is based on solid evidence and not on experimental theories.

## CONCLUSION

This manuscript describes a successful first instance of forward deployment of FSGs to an austere environment. FSG, in this military context, has shown great portability, with easy transfer of knowledge. More importantly, management with fish skin has shown faster granulation rates in burn wounds for skin grafting, resulting in improved patient outcomes with no documented infections.^11^ The concept of employing fish skin in military medical facilities, such as field hospitals, should be further explored in a controlled environment, where a more detailed analysis can be performed and secondary outcomes can be assessed.

## Data Availability

The data that support the findings of this study are available on request from the corresponding author. All data are freely accessible.
